# A Simple and Robust Vector-Based shRNA Expression System Used for RNA Interference

**DOI:** 10.1371/journal.pone.0056110

**Published:** 2013-02-06

**Authors:** Xue-jun Wang, Ying Li, Hai Huang, Xiu-juan Zhang, Pei-wen Xie, Wei Hu, Dan-dan Li, Sheng-qi Wang

**Affiliations:** Department of Biotechnology, Beijing Institute of Radiation Medicine, Beijing, People's Republic of China; George Mason University, United States of America

## Abstract

**Background:**

RNA interference (RNAi) mediated by small interfering RNAs (siRNAs) or short hairpin RNAs (shRNAs) has become a powerful genetic tool for conducting functional studies. Previously, vector-based shRNA-expression strategies capable of inducing RNAi in viable cells have been developed, however, these vector systems have some disadvantages, either because they were error-prone or cost prohibitive.

**Results:**

In this report we described the development of a simple, robust shRNA expression system utilizing 1 long oligonucleotide or 2 short oligonucleotides for half the cost of conventional shRNA construction methods and with a>95% cloning success rate. The shRNA loop sequence and stem structure were also compared and carefully selected for better RNAi efficiency. Furthermore, an easier strategy was developed based on isocaudomers which permit rapid combination of the most efficient promoter-shRNA cassettes. Finally, using this method, the conservative target sites for hepatitis B virus (HBV) knockdown were systemically screened and HBV antigen expression shown to be successfully suppressed in the presence of connected multiple shRNAs both *in vitro* and *in vivo*.

**Conclusion:**

This novel design describes an inexpensive and effective way to clone and express single or multiple shRNAs from the same vector with the capacity for potent and effective silencing of target genes.

## Introduction

RNA interference (RNAi), mediated by double-stranded RNA (dsRNA), is a natural cellular process associated with gene regulation[1]. Since the discovery of RNAi, scientists could use this instrument to deplete almost any of their interested genes not only as a tool in biological research but also as a therapeutic approach. RNAi uses a sequence-specific gene-silencing mechanism and is ultramost powerful. The general RNAi process could be artificially divided into 3 steps. First, short dsRNAs (∼20–30 nucleotides) are generated by RNase III enzymes (either Dicer alone or a combination of Drosha and Dicer); second, these short dsRNAs are unwound and the strand with a thermodynamically less stable 5′ end is preferentially loaded into the RNA-induced silencing complex (RISC) as the guide strand; third, RISC finds potential target RNAs and Argonaute (the key component of RISC) finally cleaves the target RNA containing a sequence homologous to the guide strand.

There are many ways to induce RNAi for gene knockdown experiments [2], but the two most commonly used methods are chemically synthesized siRNAs or vector-based shRNAs. Compared to chemically synthesized siRNAs, vector-based shRNA expression achieves more sustained loss of function effect especially when it is embedded in the lentiviral vector. One of the widely used shRNA expression vectors is pSuper described in 2002 [3]. It uses Pol III promoter H1 to transcribe a shRNA with a 21 bp (base pair) stem and 9 nt (nucleotide) loop structure. Furthermore, the first widely used genome-wide shRNA library utilized a lentiviral vector named pLKO.1-puro and this library has generated much of data that allowed for a better understanding of the diverse cellular processes associated with virology and cancer [4]–[7]. We observed that the pLKO.1-puro vector possessed a unique palindromic loop (CTCGAG) different from other shRNA expression vectors such as pSuper [5]. This observation resulted in the hypothesis that a shRNA structure could be constructed using only a single long or two short oligonucleotides. We further describe a strategy for rapid cloning of multiple shRNAs which permits easier combination of the most efficient promoter-shRNA cassettes for the simultaneous knockdown of multiple genes or different targets of the same gene [8], [9].

Here we gave proofs that our thought was feasible, and a shRNA could be constructed by only 1 long oligonucleotide or 2 short oligonucleotides with half the cost of conventional shRNA clone methods. Various parameters for the design of effective shRNAs based on our strategy were compared such as the palindromic loop sequences. Finally, the loop sequence “TTCTAGAA” was selected for shRNAs construction and then we gave examples that our method could apply to other genes such as the bacterial enzyme β-galactosidase (LacZ). This method was also used to successfully inhibit hepatitis B virus (HBV) antigen expression both *in vitro* and *in vivo*. This approach is cost effective and more easily applied to many areas of basic or applied research utilizing RNAi technology.

## Results

### Construction of the shRNA vector

**Figure 1:**
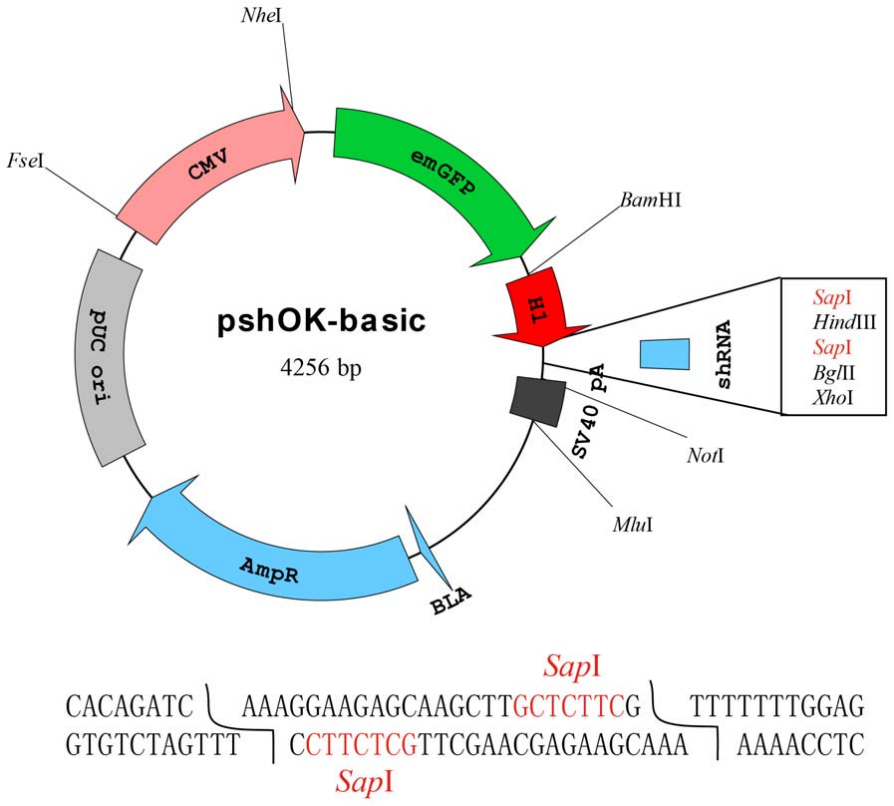
The pshOK-basic plasmid map for generation of shRNAs. The modified H1 promoter containing an AAA terminus followed by 2 *Sap* I restriction sites and 7 polyTs was used to introduce and express shRNAs. Upstream of the H1 promoter, the CMV-emGFP unit was used to tract shRNA expression. The isoaudamers *Bam* HI and *Bgl* II restriction sites were inserted as a means of generating linked shRNAs.

The plasmid named pshOK-basic was constructed (Fig. 1). Firstly, the CMV-mWasabi sequence of pmWasabi-N vector was replaced by a CMV-emGFP cassette for introduction of several new restriction sites. Then a modified H1 promoter (the terminal nucleotides changed to AAA) followed by 7 T residues in a row was directly cloned downstream of emGFP. After digested with the type IIs restriction enzyme *Sap* I, both ends of the vector would have the same TTT overhangs, thereby facilitating one of the key requirements needed to carry out the single long or 2 short oligonucleotide based shRNA cloning methods. During vector construction, every clone product was verified by restriction digestion and sequence analysis (Data S1).

### Optimization of the palindromic shRNA scaffold

**Figure 2:**
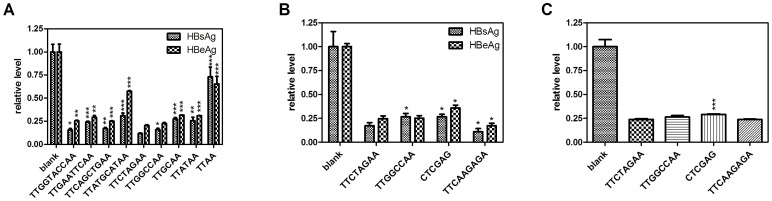
The effects of various loop sequences on shRNA silencing activity. (A) An shRNA scaffold targeted to the HBV conserved sequence “GGUAUGUUGCCCGUUUGUCCU” reported previously was selected and designed as an antisense-loop-sense structure (AS). (B) (C) The two best loops were selected and compared with two well-known loops TTCAAGAGA (used in pSuper) and CTCGAG (used in pLKO.1-puro) for two irrelevant target depression. The HBV target sequence “GGUAUGUUGCCCGUUUGUCCU” and the Gluc target sequence “UCUGUUUGCCCUGAUCUGCAU” were used in (B) and (C) respectively. Statistical significance was determined respectively by comparing shRNAs groups with that containing “TTCTAGAA” loop. Means and standard deviations were generated from 3 independent experiments. The “blank” group represents cells treated with pshOK-basic instead of the shRNA plasmid. The value in the blank group was set at 1.0.

Another critical component to the successful cloning of shRNA using the methods described is that the loop sequence needs to be palindromic. We constructed a series of shRNAs with different lengths of palindromic loops to target a defined HBV conserved sequence (GGUAUGUUGCCCGUUUGUCCU). Here, the HBV conserved target sequence was selected as the target because its antigen products (HBsAg and HBeAg) are secreted into the supernatants thereby facilitating their detection using commercial kits. This target sequence was conserved across different HBV genotypes and thoroughly demonstrated by several groups reported previously [10], [11]. All the shRNAs had an antisense-sense (AS) stem structure. As shown in Fig. 2A, the shRNAs with loops containing 6, 8, and 10 nucleotides all had above a 70% knockdown efficiency against HBsAg and HBeAg except for TTATGCATAA. Otherwise, when the loop sequence was shortened to 4 nucleotides, the inhibition rate dropped below 50% indicating that the nucleotide size of the loop should be above 4. The 8 nucleotide loops demonstrated the highest level of gene down regulation, especially for TTCTAGAA and TTGGCCAA. Then the shRNAs knockdown efficiency of the TTCTAGAA and TTGGCCAA loops was compared with the well-established loops TTCAAGAGA (used in pSuper) and CTCGAG (used in pLKO.1-puro) for two irrelevant target depression. These results were shown in Fig. 2B and Fig. 2C. It was indicated that the shRNAs with TTCTAGAA loop were superior than CTCGAG (used in pLKO.1-puro, Fig. 2B and 2C) but inferior to TTCAAGAGA (used in pSuper, Fig. 2B). Overall, the 8-nt loop “TTCTAGAA” gave us a usable and relatively better silencing activity among the detected palindromic loops. We therefore selected this sequence as our shRNA scaffold loop for subsequent experiments.

In a previous report that used the pSuper vector, shRNA efficiency was also influenced by the position of the antisense and sense strands [12]. We therefore cloned an shRNA named SA1856 with an sense-antisense (SA) stem also containing the TTCTAGAA loop and assessed its ability to inhibit HBsAg and HBeAg expression compared to the AS isoform. In 3 independent experiments, we did not identify discernible differences in the inhibition rates between these constructs, but the other two shRNAs with different stem structures targeting another HBV sequence “CACUGUUUGGCUUUCAGUUAU” [12] showed a different result (Fig. S1). It revealed that the shRNA with antisense-sense stem may have a relatively better activity than the shRNA with sense-antisense stem, although more evidence is needed. Considering that some of the loop sequence may be contributed to the stem portion of the shRNA molecule, the shRNAs with the antisense-sense stem were selected and recommended.

### Two short oligonucleotides based shRNA construction method is superior to the single long oligonucleotide based strategy

**Figure 3:**
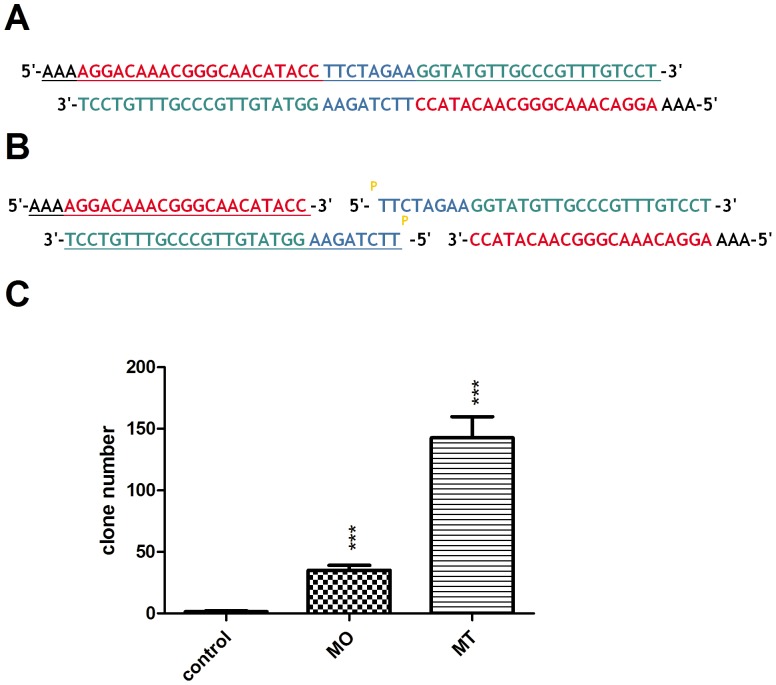
Comparison of the two shRNA construction methods. (A) The shRNA clone method based on one long oligonucleotide (MO). The oligo underlined was synthesized and annealed to its self to form double strands. (B) The shRNA clone method based on two short oligonucleotides (MT). Two short oligonucleotides (underlined) were synthesized and the 5'-end of the oligo containing the loop sequence (TTCTAGAA) phosphorylated by the T4 polynucleotide kinase in the presence of ATP. Then, the two short oligonucleotides were annealed to form double strands. (C) The shRNA cloning efficiency of the two methods was compared. The vector pshOK-basic was digested with *Sap* I and ligated with the annealed double strand oligos as described above. The “control” group represents the linearized pshOK-basic ligated in the absence of oligos. Means and standard deviations were generated from 3 independent experiments.

We designed two ways to construct the palindromic shRNA scaffold. One route was that a single long oligonucleotide was synthesized and annealed to double strands by itself. The other way was that two short oligonucleotides were synthesized and 5'-end of the oligo containing the loop sequence was phosphorylated by T4 polynucleotide kinase in the presence of ATP. Then the two short oligonucleotides were annealed to double strands as shown in Fig. 3B.

The pshOK-basic vector was digested by *Sap* I to generate two TTT overhangs and then ligated with the double strands oligos described above. The ligation efficiency of the two methods was compared and obviously the two short oligonucleotides based shRNA construction method was superior to the single long oligonucleotide based strategy as shown in Fig. 3C. Three clones were picked, digested and sequenced respectively to confirm that the cloning procedures did not introduce any changes to the sequences. Considering that the short oligo had a theoretically lower synthetic error rate and was more cost-effective than the longer one, the two short oligonucleotide based shRNA construction method was chosen as our primary method. But the clone steps seemed more complex, so the single long oligonucleotide based strategy was also an alternative way for your choice.

### Effective shRNA-mediated suppression of two reporter genes

**Figure 4:**
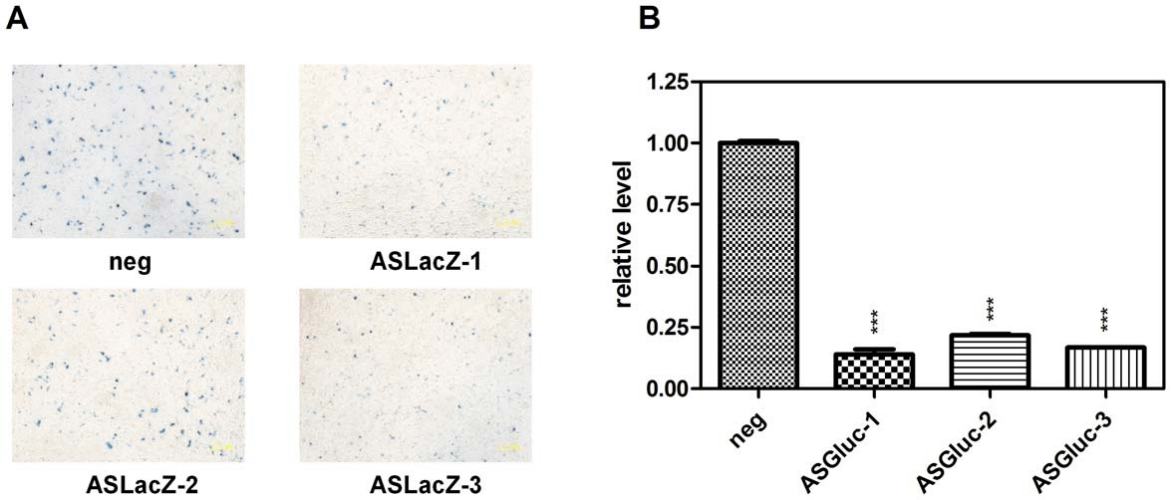
Suppression of two reporter genes by the shRNAs cloned with our methods. (A) HepG2 cells were seeded in 24-well plates and cotransfected with 200 ng of pAAV-LacZ, 200 ng shRNA plasmid and 100 ng pSEAP2-Control (used as a normalization control). LacZ was stained and photographed 48 h after cotransfection of HepG2 cells. Magnification ×200. The scale bar represents 1 µm. (B) The same procedure described above was carried out using pCMV-Gluc in place of pAAV-LacZ. After 48 h, Gluc activity was determined. An shRNA scaffold (targeted to GUCUCCACGCGCAGUACAUUU) irrelevant to any known human or mouse gene sequence was designed as the negative control (“neg”) [23], [24]. Means and standard deviations were generated from 3 independent experiments.

To thoroughly test the efficiency of our shRNA scaffold to elicit RNAi activity, we selected 2 reporter genes, LacZ and the secretory *Gaussia princeps* luciferase (Gluc) for evaluation. Three shRNAs targeting each gene were designed and constructed, respectively (Table 1). HepG2 cells were cotransfected with shRNA, the target gene and the normalization control vector pSEAP2-Control. After a 48 h transfection, LacZ and Gluc expression was detected. To our surprise, 5 out of 6 shRNAs gave a satisfactory knockdown rate except ASLacZ-2 (Fig. 4). We imagine that the ASLacZ-2 target neighbouring sequence may have a higher structure and hinder siRNA to access it. Whatever, these results were very encouraging. We moved on to test whether our strategy was also practical for disease treatment.

**Table 1:** Target sequences of the shRNAs.

shRNA name	Target sequence (5'-3')
ASLacZ-1	GCAGUUAUCUGGAAGAUCAGG
ASLacZ-2	UGGCAGGCGUUUCGUCAGUAU
ASLacZ-3	CGGCGACUUCCAGUUCAACAU
ASGluc-1	UCUGUUUGCCCUGAUCUGCAU
ASGluc-2	UGCCUUCGUGCAGUGUUCUGA
ASGluc-3	UGCGACCUUUGCCAGCAAGAU
AS139	UGCCUUCUGACUUCUUUCCUU
AS618	CGGGAAUCUCAAUGUUAGUAU
AS1819	GCUGCUAUGCCUCAUCUUCUU
AS1850	UACCAAGGUAUGUUGCCCGUU
AS1856	GGUAUGUUGCCCGUUUGUCCU
AS2056	CCGUUUCUCCUGGCUCAGUUU
AS2068	GCUCAGUUUACUAGUGCCAUU
AS2090	GUUCAGUGGUUCGUAGGGCUU
AS2497	UCGCCAACUUACAAGGCCUUU
AS3002	UCGCAUGGAAACCACCGUGAA
AS3083	AACGACUGACCUUGAGGCAUA
AS3172	UAGGAGGCUGUAGGCAUAAAU

### Effect of shRNAs on hepatitis B virus infection in vitro and in vivo

**Figure 5:**
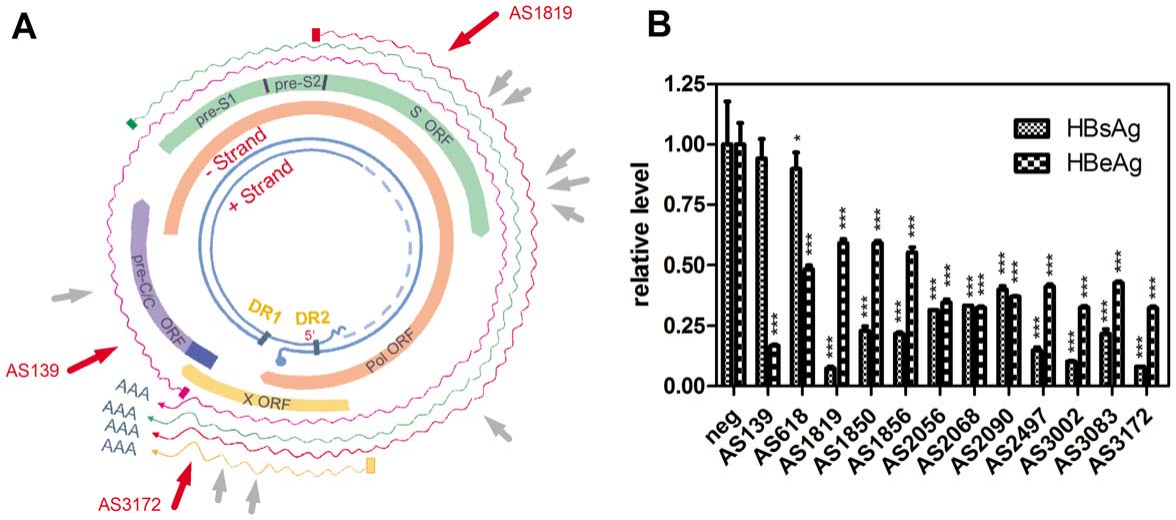
Screening of shRNAs for significant suppression of HBsAg and HBeAg. (A) shRNAs targeting to the conserved regions of HBV genome were designed and illustrated. The numbers represent nucleotide (nt) coordinates relative to the HBV (genotype B) pgRNA start site. (B) HepG2 cells were seeded in 24-well plates and cotransfected with 200 ng of pHBV1.31, 200 ng shRNA plasmid and 100 ng pSEAP2-Control per well. The HBsAg and HBeAg concentrations in cell supernatants were detected 48 h post transfection. Means and standard deviations were generated from 3 independent experiments.

**Figure 6:**
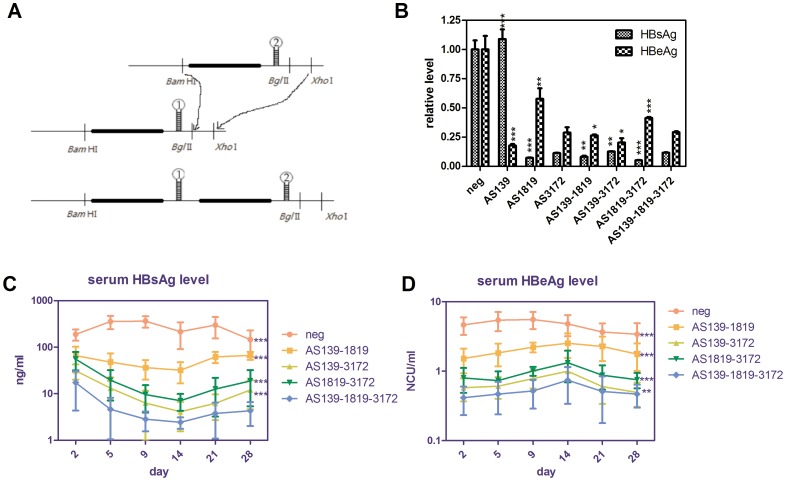
Efficient down regulation of HBsAg and HBeAg expression using linked shRNAs concatemers both *in vitro* and *in vivo*. (A) The diagram illustrates the principle of chaining two shRNAs derived from two different shRNA vectors into one vector. (B) HepG2 cells were seeded in 24-well plates and cotransfected with 200 ng of pHBV1.31, 200 ng shRNA plasmid and 100 ng pSEAP2-Control (as a normalization control) per well. After 48 h, the HBsAg and HBeAg concentrations were determined. Means and standard deviations were generated from 3 independent experiments. (C) Serum HBsAg and (D) HBeAg were measured by quantitative ELISA at the indicated days after plasmids delivery. Groups of male C57/BL6 mice (n = 6) were intravenously injected with 6 µg pHBV1.18, 3 µg shRNA plasmids and 3 µg pSEAP2-Control (as an internal control). NCU is the abbreviation of “National Clinical Unit”. Statistical significance was determined respectively by comparing shRNAs groups with AS139-1819-3172. Due to limited serum resources, each sample was diluted 20-fold.

We selected hepatitis B virus as the test target which is an important infectious disease in China. First, we screened 12 shRNAs targeted to different conserved HBV sequences resulting in the identification of several highly efficient shRNAs (Table 1). Among them, AS1819 targeted to the HBsAg ORF was the most potent inhibitor of HBsAg expression, while AS139 targeted to the HBc/HBe ORF had the most potent inhibition rate for HBeAg expression. AS3172 targeted to the HBxAg ORF had potent inhibition rate both for HBsAg and HBeAg expression (Fig. 5B). RT-PCR and ELISA experiments showed that AS139 inhibited pc/pgRNA and HBeAg expression dose-dependently (Fig. S2 and S3). We next connected these shRNAs by sub-cloning and compared different combination knockdown efficiencies both *in vitro* and *in vivo*. As illustrated in Fig. 6B, the combination of AS139, AS1819 and AS3172 resulted in the efficient suppression of HBV antigen expression *in vitro* and the best inhibition rate *in vivo* (above 90%, Fig. 6C and 6D).

## Discussion

In this study, we have constructed a shRNA vector with several characteristics for its better and easier use. First, we employed the H1 RNA polymerase III promoter instead of U6 whose toxicity has been previously identified [13]. The Pol III H1 promoter has a well-defined transcription start site proven to be more flexible than the U6 promoter with regard to +1 sequence changes. Second, proper isocaudomers (*Bam* HI and *Bgl* II) were used for easier cascade connected shRNAs construction. Third, the CMV-emGFP cassette was used to track shRNA transfection. This cassette could be replaced easily by other therapeutic genes as a means of overexpressing one gene while concomitantly knocking down another gene. The resulting vector was named pshOK-basic (overexpression and knockdown).

For efficient and lower-cost shRNA construction, several routes was reported previously including the one-oligonucleotide method combined with PCR [14] or the four short oligonucleotides based strategy [15]. The most significant advantage to the method described in this study is that only single long (without PCR) or two short oligonucleotides were required for cloning shRNAs. To accomplish this goal, a unique palindromic shRNA scaffold was screened and optimized. The terminal sequence of the H1 promoter was first changed to AAA for shRNA clone with restriction enzyme *Sap* I. Next, several shRNAs with different palindromic loops were cloned, compared and carefully selected based on their respective knockdown efficiencies. Previously, various loop sequences were used and systematically investigated by different investigators, but these results were inconsistent [16], [17]. In general, the shRNAs with a relative long open loop sequence would have greater silencing activity than the corresponding shRNA with a shorter complementary loop sequence. But the shRNAs with only a 3-nt loop were also reported for their good knockdown efficiency, and the well-known pLKO.1-puro vector possessed a unique 6-nt palindromic loop (CTCGAG). In this study, we demonstrated that most of shRNAs with a more than 4-nt palindromic loop would have a relative good silencing activity. The inferior performance of shRNAs with a 4-nt loop may be due to their inefficient processing by Dicer [17]. Due to its palindromic nature, there may be some contribution of this loop sequence to the stem portion of the shRNA molecule. Thus if the sense-loop-antisense shRNAs were designed by our method, the 5'-terminal of the antisense sequence would be changed by Dicer procession and possibly interfere with the target recognition. In addition, according to the latest research progress, the imperfect specificity of Dicer itself was also contributed to the miRNA and siRNA length heterogeneity [18], [19]. This was part of the reasons why the antisense-loop-sense shRNA scaffold was chosen in this paper. In addition, this shRNA system was also compared with the popular used vector pSuper and the results indicated that a relative long and open loop would be an important factor that influenced shRNA silencing activity. But the stem structure of pSuper vector also influenced its silencing activity (Fig. S4). Overall, the new shRNA system demonstrated here gave us an acceptable and more economical way to knockdown genes. At the last, two different shRNA clone methods were compared and the two short oligonucleotides based shRNA construction method was more efficient than the single long oligonucleotide based strategy. The single long oligo would be prone to form hairpin structure itself, and this could affect the double strands formation. We think this is the reason why the two short oligos method was superior to the single long oligo strategy. With shorter oligos, the error rate of synthesis was also decreased.

After successfully suppression of the LacZ and Gluc genes using the method described, we tested this approach as a means of combating HBV infections that represent an important public health threat in China. Today there are several high performance nucleotide analogs that can suppress HBV DNA replication, but there are no clinically approved drugs with the capacity of suppressing or preventing the expression of HBV antigens, especially for HBsAg. HBsAg plays important roles in the HBV life cycle and in the establishment of chronic infections [20]. Therefore, HBsAg clearance is critical to the development of successful HBV antiviral therapies. In this study, we utilized our shRNA method to successfully suppress HBsAg and HBeAg expression in the absence of detectable shRNA-related interferon responses (Fig. S5). However, we didn't observe differences in knockdown efficiency enhancement following the combination of 2 or 3 shRNAs *in vitro*, suggesting that this phenomenon might be caused by promoter disturbances or competition. An indirect experiment using the 3'-UTR luciferase assay was used to demonstrate that the silencing activity of AS139-1819-3172 were indeed some lower comparing with AS139, AS1819 and 3172 respcetively (Fig. S6). We also found that the co-transfected multiple shRNA constructs had better silencing activity than the shRNA plasmid with multiple shRNAs when the same amount of each shRNA scaffold was used (Fig. S7). This verified our speculation that multiple H1 promoter in the same vector may interference with each other. Regardless, the three connected shRNA structure was shown to efficiently inhibit HBV antigens expression especially *in vivo*.

## Conclusions

We describe a simple and robust shRNA construction system that will enable users to easily construct single or multiple shRNAs efficiently at a low cost. Using this method, we systemically screened the target sites for HBV knockdown and successfully depressed HBV antigen expression with connected multiple shRNAs both *in vitro* and *in vivo*. The method described here provides an inexpensive and powerful new tool with the potential of down regulating gene expression that can be applied to a variety of biological systems, including treatment of various diseases. Finally, it would be highly beneficial to generate an economic and high-performance lentiviral vector system based on our shRNA method to carry out high-throughput genetic screens for loss-of-function phenotypes.

## Materials and Methods

### Ethics statement

The Medical Ethics Committee of Beijing Institute of Radiation Medicine specifically approved this study. All the male C57/BL6 mice used in this experiment received humane care. The hydrodynamic injection was performed under sodium pentobarbital anesthesia, and every effort was made to minimize suffering.

### Plasmid construction

The HBV replication-competent plasmids pHBV1.31 and pHBV1.18 containing 1.31 and 1.18 HBV genome copies, respectively, were constructed and preserved in our laboratory (unpublished data), pmWasabi-N was a gift from Allele Biotechnology Company, pSuper was obtained from Addgene (Cambridge, MA), vector pSEAP2-Control was a gift from Dr. Shui-ping Liu (Central South University of China), and the pCMV-GLuc vector was purchased from New England Biolabs (NEB, Ipswich, MA). To generate the pshOK-basic vector, the CMV-mWasabi sequence present in pmWasabi-N was replaced with a CMV-emGFP cassette as a means of introducing respective restriction sites. In addition, a modified human RNA-polymerase III H1 promoter was amplified from pSuper and cloned into the pCMV-emGFP generated above. Then, 2 short oligos containing 7 polyTs were annealed in NEB buffer 2 and inserted into the above-described vector containing a modified human H1 promoter. Two strategies were used to generate shRNA. The first used a single long oligonucleotide synthesized and annealed to form double strands of its self. The second approach used two short oligonucleotides and the 5'-end of the oligo containing the loop sequence was phosphorylated by T4 polynucleotide kinase in the presence of ATP. Then these two short oligonucleotides were annealed to form double strands. Plasmid pshOK-basic was digested using *Sap* I, gel-purified and ligated with the annealed products at 16 ^°^C for 3 h using DNA Ligation Kit Ver.2.0 (Takara).

### Cell culture and transfection

The HepG2 and HEK293T cell lines were purchased from the Cell Bank of Shanghai Institute of Cell Biology (Shanghai, China) and cultured in DMEM (Dulbecco's Minimal Essential Media) medium containing 10% FBS (fetal bovine serum), 100 U/ml penicillin and 100 µg/ml streptomycin and maintained at 37 ^°^C in a humidified 5% CO_2_ atmosphere. Plasmid cotransfections were carried out 16 h after seeding the cells using GenJet™ Reagent (Ver. II) (SignaGen Laboratories, Rockville, MD) following the manufacturer's protocol.

### Reporter gene assays

For the β-gal assays, HepG2 cells were plated at 1.5×10^5^ cells per well in 24-well tissue culture plates (Nunc, Roskilde, Denmark) and cotransfected with 200 ng of pAAV-LacZ (Stratagene), 200 ng shRNA plasmid and 100 ng pSEAP2-Control as a normalization control. After 48 h, HepG2 cells were stained using the β-Galactosidase Staining Kit (Beyotime Institute of Biotechnology). For the secretory Gaussia princeps luciferase (Gluc) assay, the same procedure was used but pAAV-lacZ was replaced with pCMV-Gluc. 48 h post transfection, the culture supernatants were collected and centrifuged at 12,000×g for 5 min to remove debris. Gluc activity was measured using BioLux^®^ Gaussia Luciferase Flex Assay Kit (New England Biolabs). To monitor transfection efficiency, cell supernatants were assayed for secreted alkaline phosphatase (SEAP) using a SEAP Reporter Assay Kit (Toyobo).

### 3'-UTR Luciferase assay

The sequences containing the shRNAs target sites were cloned into a modified pGL3-control plasmid (pGL3M) as described previously[21], [22]. HEK293T cells were co-transfected with 200 ng of pGL3M-UTR constructs and 200 ng shRNAs per well in 24-well plates GenJet™ Reagent (Ver. II). 100 ng pRL-CMV (Promega, Madison, WI) was co-transfected as the normalization control. Luciferase activity assays were performed 48 h post transfection using the dual luciferase reporter assay system (Promega).

### HBsAg and HBeAg ELISA

HepG2 cells were cotransfected with 200 ng of pHBV1.31, 200 ng shRNA plasmid and 100 ng pSEAP2-Control as a normalization control per well of 24-well tissue culture plates. Media was changed and collected 48 h after HBV plasmid transfection and supernatants centrifuged at 12,000×g for 5 min to remove debris before collection. The concentrations of HBsAg and HBeAg in the supernatants were determined by chemiluminescence using commercial assay kits (Tigsun Diagnostics Co., Ltd., Beijing, China).

### Transfection with shRNA vectors does not induce an interferon response

HepG2 cells were transfected with the shRNA vectors and untransfected cells were treated with 1000 IU of IFNα-2a (Shenyang Sunshine Pharmaceutical Company) for 24 h or left untreated. Total cellular RNA was prepared using TRIZOL (Invitrogen, Carlsbad, CA). Semi-quantitative RT-PCR using a 2-step method was used to determine the mRNA expression level of several interferon inducible genes. PCR products were analyzed by agarose gel electrophoresis followed by ethidium bromide staining. Primers for α-actin, and the IFN-inducible genes MxA, 2', 5'-oligoadenylate synthetase 1 (OAS1), signal transducer, activator of transcription 1 (STAT1), and interferon-stimulated gene 15 (ISG15) are described in reference [12].

### Animal and hydrodynamic transfection

Male C57/BL6 mice weighing 16–18 g (4–5 weeks old at the start of the experiments) were obtained and housed in the animal center of the Academy of Military Medicine Science. To evaluate the anti-viral effects of shRNAs *in vivo*, an HBV hydrodynamic injection was conducted. Briefly, purified HBV plasmid pHBV1.18 (6 µg), shRNA plasmids (3 µg) and the pSEAP2-Control (3 ug) as an internal control were diluted in physiological saline in a volume equivalent to 10% of the body weight and then injected into the tail vein within 5–8 s. Sera was then assayed for HBsAg and HBeAg. For each group, six mice were used. All animals received humane care and the study protocol complied with the institution's ethics guidelines.

### Statistical analysis

The data presented here were expressed as mean ± standard deviation (SD) and statistical significance was determined by the Student's t test or two-way ANOVA. P-values are indicated by asterisks (***P<0.001, **P<0.01, *P<0.05).

## Supporting Information

Figure S1 **The effects of stem structures on shRNA silencing activity.** The antisense-sense (AS) shRNAs and the sense-antisense (SA) shRNAs were compared with each other for their anti-HBV activity. The HBV target sequences “GGUAUGUUGCCCGUUUGUCCU” (1856) and “CACUGUUUGGCUUUCAGUUAU” (2116) were used in (A) and (B) respectively.
(TIF)

Figure S2 **Transfection with shRNA vector AS139 inhibited HBV pc/pgRNA dose-dependently.** Primers HBVF ( CGTTTTTGCCTTCTGACTTCTTTC ) and HBVR (ATAAGATAGGGGCATTTGGTGGTC) were used for HBV pc/pgRNA amplification. Lane 1, pshOK-basic:pHBV1.31 = 1∶1; lane 2, pshOK-neg:pHBV1.31 = 1∶1; lane 3, pshOK-neg:AS139: pHBV1.31 = 3∶1∶4; lane 4, pshOK-neg:AS139: pHBV1.31 = 1∶1∶2; lane 5, AS139: pHBV1.31 = 1∶1.
(TIF)

Figure S3 **Transfection with shRNA vector AS139 inhibited HBeAg antigen dose-dependently.** HepG2 cells were seeded in 24-well plates and cotransfected with 200 ng of pHBV1.31, 200 ng shRNA plasmid (AS139 plus neg) and 100 ng pSEAP2-Control per well. The HBsAg and HBeAg concentrations in cell supernatants were detected 48 h post transfection. Means and standard deviations were generated from 3 independent experiments.
(TIF)

Figure S4 **This pshOK shRNA system was compared with the popular used vector pSuper.** The HBV target sequence “GGUAUGUUGCCCGUUUGUCCU” and the Gluc target sequence “UCUGUUUGCCCUGAUCUGCAU” were used in (A) and (B) respectively. Statistical significance was determined by comparing to shRNAs groups AS1856 and ASGluc-1 respectively.
(TIF)

Figure S5 **Transfection with shRNA vectors didn't induce an obvious interferon response.** Lane 1, mock; lane 2, IFNα-2a; lane 3, AS139; lane 4, AS1819; lane 5, AS3172; lane 6, neg.
(TIF)

Figure S6 **An indirect experiment using the 3'-UTR luciferase assay to demonstrate that these shRNAs were expressed.** The relative luciferase level of AS139-1819-3172 was compared with AS139, AS1819 and AS3172 respectively.
(TIF)

Figure S7 **The co-transfected multiple shRNA constructs had better silencing activity than the shRNA plasmid with multiple shRNAs when the same amount of each shRNA scaffold was used.**
(TIF)

Data S1 **The typical sequencing data covering the shRNA expression element, the full pshOK-basic plasmid sequence and their alignment file.**
(ZIP)

## References

[1] Meister G, Tuschl T (2004) Mechanisms of gene silencing by double-stranded RNA. Nature 431: 343–349. 15372041 10.1038/nature02873

[2] Sibley CR, Seow Y, Wood MJ (2010) Novel RNA-based strategies for therapeutic gene silencing. Molecular therapy: the journal of the American Society of Gene Therapy 18: 466–476. 20087319 10.1038/mt.2009.306 PMC2839433

[3] Brummelkamp TR, Bernards R, Agami R (2002) A system for stable expression of short interfering RNAs in mammalian cells. Science 296: 550–553. 11910072 10.1126/science.1068999

[4] Khurana A, Tun HW, Marlow L, Copland JA, Dredge K, et al (2012) Hypoxia negatively regulates heparan sulfatase 2 expression in renal cancer cell lines. Mol Carcinog 51: 565–575. 21739484 10.1002/mc.20824 PMC3192919

[5] Moffat J, Grueneberg DA, Yang X, Kim SY, Kloepfer AM, et al (2006) A lentiviral RNAi library for human and mouse genes applied to an arrayed viral high-content screen. Cell 124: 1283–1298. 16564017 10.1016/j.cell.2006.01.040

[6] Root DE, Hacohen N, Hahn WC, Lander ES, Sabatini DM (2006) Genome-scale loss-of-function screening with a lentiviral RNAi library. Nat Methods 3: 715–719. 16929317 10.1038/nmeth924

[7] Chen YC, Su WC, Huang JY, Chao TC, Jeng KS, et al (2010) Polo-like kinase 1 is involved in hepatitis C virus replication by hyperphosphorylating NS5A. Journal of virology 84: 7983–7993. 20534861 10.1128/JVI.00068-10 PMC2916529

[8] Xu XM, Yoo MH, Carlson BA, Gladyshev VN, Hatfield DL (2009) Simultaneous knockdown of the expression of two genes using multiple shRNAs and subsequent knock-in of their expression. Nature protocols 4: 1338–1348. 19713955 10.1038/nprot.2009.145 PMC2753455

[9] Chumakov SP, Kravchenko JE, Prassolov VS, Frolova EI, Chumakov PM (2010) Efficient downregulation of multiple mRNA targets with a single shRNA-expressing lentiviral vector. Plasmid 63: 143–149. 20064551 10.1016/j.plasmid.2009.12.003 PMC2849729

[10] Chen CC, Ko TM, Ma HI, Wu HL, Xiao X, et al (2007) Long-term inhibition of hepatitis B virus in transgenic mice by double-stranded adeno-associated virus 8-delivered short hairpin RNA. Gene therapy 14: 11–19. 16929350 10.1038/sj.gt.3302846

[11] Wu HL, Huang LR, Huang CC, Lai HL, Liu CJ, et al (2005) RNA interference-mediated control of hepatitis B virus and emergence of resistant mutant. Gastroenterology 128: 708–716. 15765406 10.1053/j.gastro.2004.12.007 PMC7094679

[12] Sun D, Rosler C, Kidd-Ljunggren K, Nassal M (2010) Quantitative assessment of the antiviral potencies of 21 shRNA vectors targeting conserved, including structured, hepatitis B virus sites. J Hepatol 52: 817–826. 20400195 10.1016/j.jhep.2009.10.038

[13] Grimm D, Streetz KL, Jopling CL, Storm TA, Pandey K, et al (2006) Fatality in mice due to oversaturation of cellular microRNA/short hairpin RNA pathways. Nature 441: 537–541. 16724069 10.1038/nature04791

[14] Flores-Jasso CF, Velazquez-Quesada I, Landa-Solis C, Gutierrez AA, Vaca L (2005) One-oligonucleotide method for constructing vectors for RNA interference. Acta Pharmacol Sin 26: 1467–1473. 16297345 10.1111/j.1745-7254.2005.00230.x

[15] Li XX, Jia HW, Quan JX, Jiao YL, Yang YH, et al (2008) An efficient approach for constructing shRNA expression vectors based on short oligonucleotide synthesis. Analytical biochemistry 381: 163–165. 18601895 10.1016/j.ab.2008.06.011

[16] Miyagishi M, Sumimoto H, Miyoshi H, Kawakami Y, Taira K (2004) Optimization of an siRNA-expression system with an improved hairpin and its significant suppressive effects in mammalian cells. The journal of gene medicine 6: 715–723. 15241778 10.1002/jgm.556

[17] Li L, Lin X, Khvorova A, Fesik SW, Shen Y (2007) Defining the optimal parameters for hairpin-based knockdown constructs. RNA 13: 1765–1774. 17698642 10.1261/rna.599107 PMC1986814

[18] Starega-Roslan J, Krol J, Koscianska E, Kozlowski P, Szlachcic WJ, et al (2011) Structural basis of microRNA length variety. Nucleic acids research 39: 257–268. 20739353 10.1093/nar/gkq727 PMC3017592

[19] McIntyre GJ, Yu YH, Lomas M, Fanning GC (2011) The effects of stem length and core placement on shRNA activity. BMC molecular biology 12: 34. 21819628 10.1186/1471-2199-12-34 PMC3175162

[20] Xu Y, Hu Y, Shi B, Zhang X, Wang J, et al (2009) HBsAg inhibits TLR9-mediated activation and IFN-alpha production in plasmacytoid dendritic cells. Molecular immunology 46: 2640–2646. 19501403 10.1016/j.molimm.2009.04.031

[21] Cui J, Fu H, Feng J, Zhu J, Tie Y, et al (2007) The construction of miRNA expression library for human. Prog Biochem Biophys 34: 389–394.

[22] Hu W, Wang X, Ding X, Li Y, Zhang X, et al (2012) MicroRNA-141 represses HBV replication by targeting PPARA. PloS one 7: e34165. 22479552 10.1371/journal.pone.0034165 PMC3316618

[23] Chen A, Dong L, Leffler NR, Asch AS, Witte ON, et al (2011) Activation of GPR4 by acidosis increases endothelial cell adhesion through the cAMP/Epac pathway. PloS one 6: e27586 . 22110680 10.1371/journal.pone.0027586 PMC3217975

[24] McLaughlin J, Cheng D, Singer O, Lukacs RU, Radu CG, et al (2007) Sustained suppression of Bcr-Abl-driven lymphoid leukemia by microRNA mimics. Proceedings of the National Academy of Sciences of the United States of America 104: 20501–20506. 18079287 10.1073/pnas.0710532105 PMC2154460

