# Supplementary figures and images for: A Simple and Robust Vector-Based shRNA Expression System Used for RNA Interference
Source: PLoS One. 2013 Feb 6;8(2):e56110. doi: 10.1371/journal.pone.0056110 (PMC3566080; doi:10.1371/journal.pone.0056110)

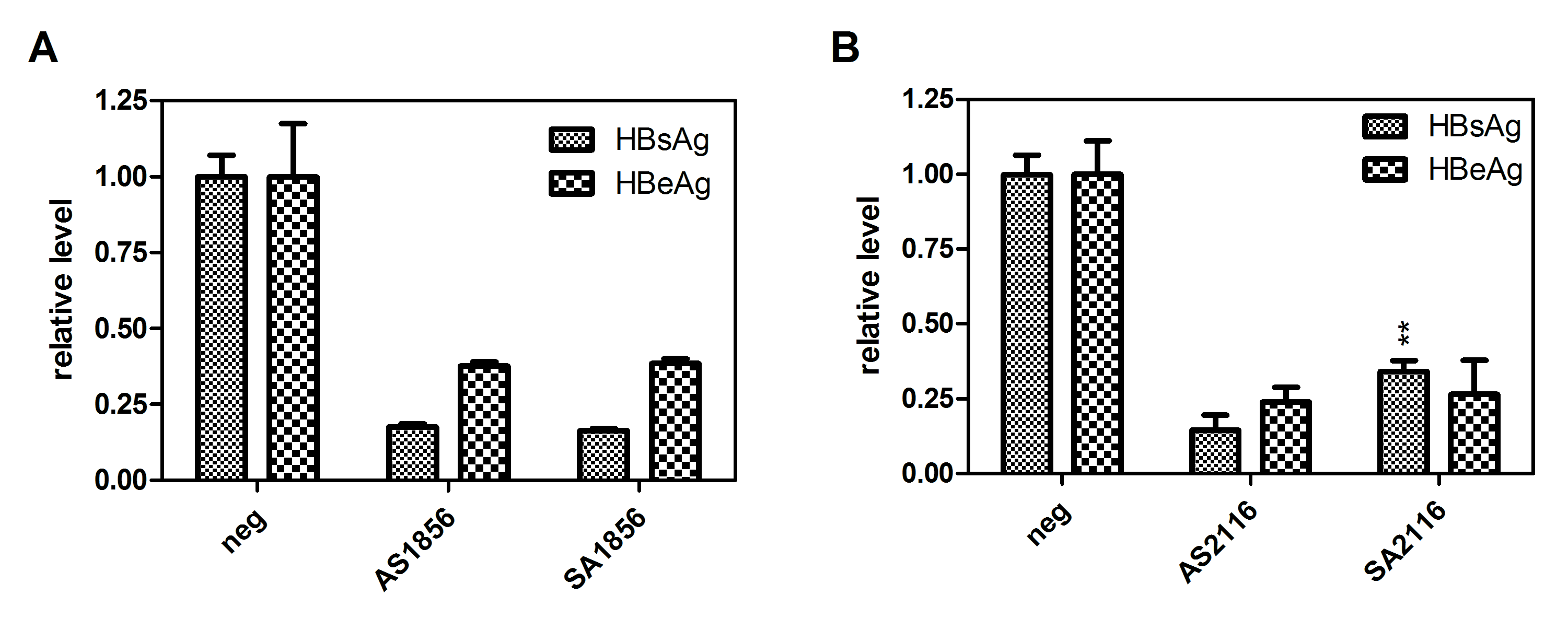

Supplement: Figure S1 — The effects of stem structures on shRNA silencing activity. The antisense-sense (AS) shRNAs and the sense-antisense (SA) shRNAs were compared with each other for their anti-HBV activity. The HBV target sequences “GGUAUGUUGCCCGUUUGUCCU” (1856) and “CACUGUUUGGCUUUCAGUUAU” (2116) were used in (A) and (B) respectively. (TIF) [file pone.0056110.s001.tif]

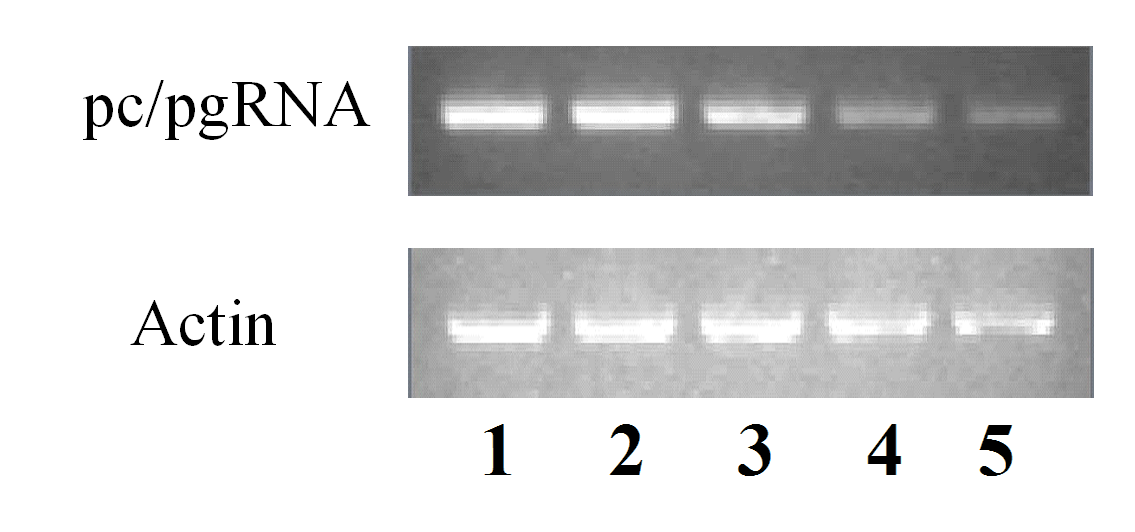

Supplement: Figure S2 — Transfection with shRNA vector AS139 inhibited HBV pc/pgRNA dose-dependently. Primers HBVF ( CGTTTTTGCCTTCTGACTTCTTTC ) and HBVR (ATAAGATAGGGGCATTTGGTGGTC) were used for HBV pc/pgRNA amplification. Lane 1, pshOK-basic:pHBV1.31 = 1∶1; lane 2, pshOK-neg:pHBV1.31 = 1∶1; lane 3, pshOK-neg:AS139: pHBV1.31 = 3∶1∶4; lane 4, pshOK-neg:AS139: pHBV1.31 = 1∶1∶2; lane 5, AS139: pHBV1.31 = 1∶1. (TIF) [file pone.0056110.s002.tif]

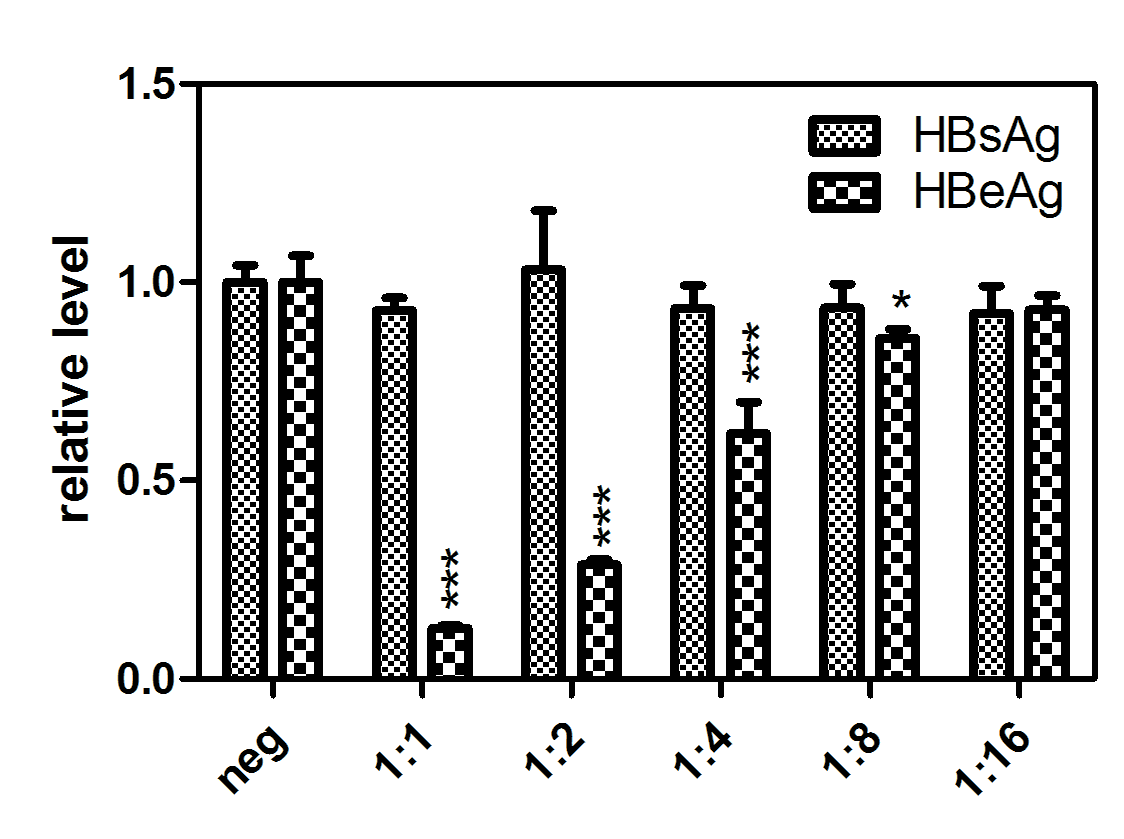

Supplement: Figure S3 — Transfection with shRNA vector AS139 inhibited HBeAg antigen dose-dependently. HepG2 cells were seeded in 24-well plates and cotransfected with 200 ng of pHBV1.31, 200 ng shRNA plasmid (AS139 plus neg) and 100 ng pSEAP2-Control per well. The HBsAg and HBeAg concentrations in cell supernatants were detected 48 h post transfection. Means and standard deviations were generated from 3 independent experiments. (TIF) [file pone.0056110.s003.tif]

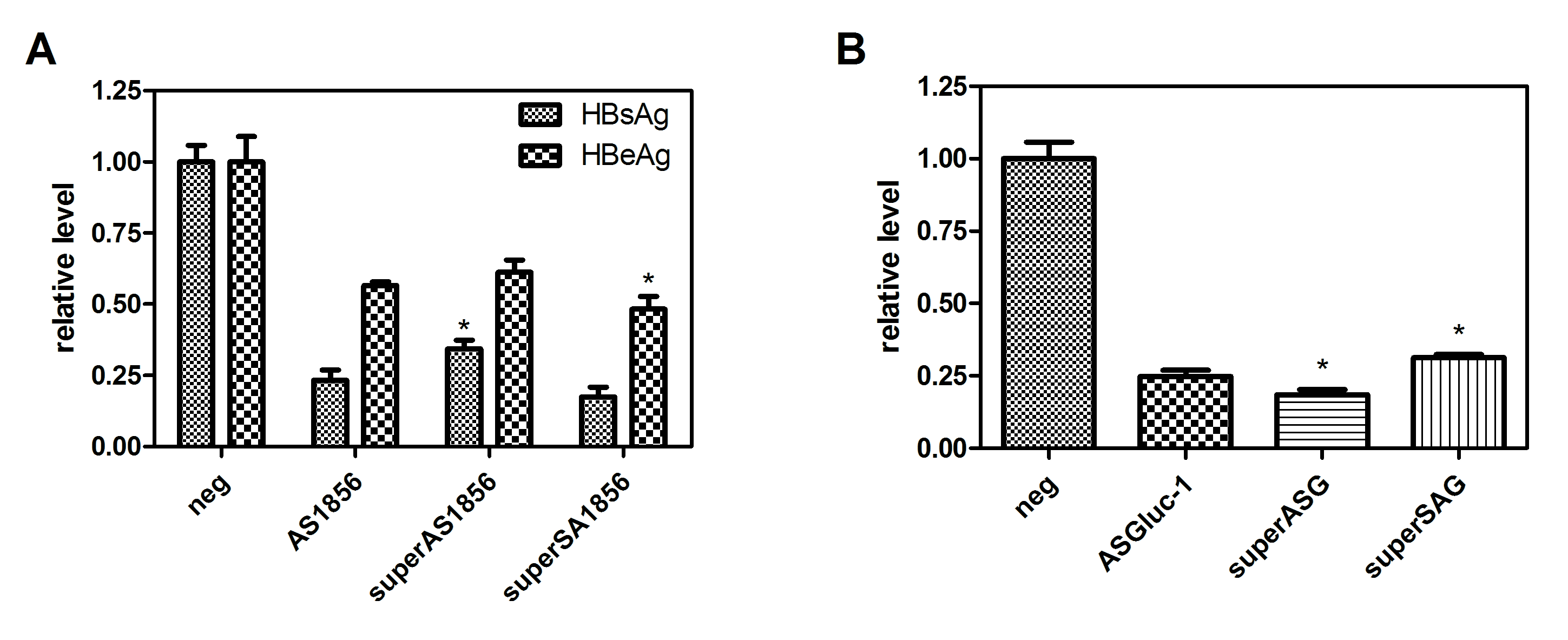

Supplement: Figure S4 — This pshOK shRNA system was compared with the popular used vector pSuper. The HBV target sequence “GGUAUGUUGCCCGUUUGUCCU” and the Gluc target sequence “UCUGUUUGCCCUGAUCUGCAU” were used in (A) and (B) respectively. Statistical significance was determined by comparing to shRNAs groups AS1856 and ASGluc-1 respectively. (TIF) [file pone.0056110.s004.tif]

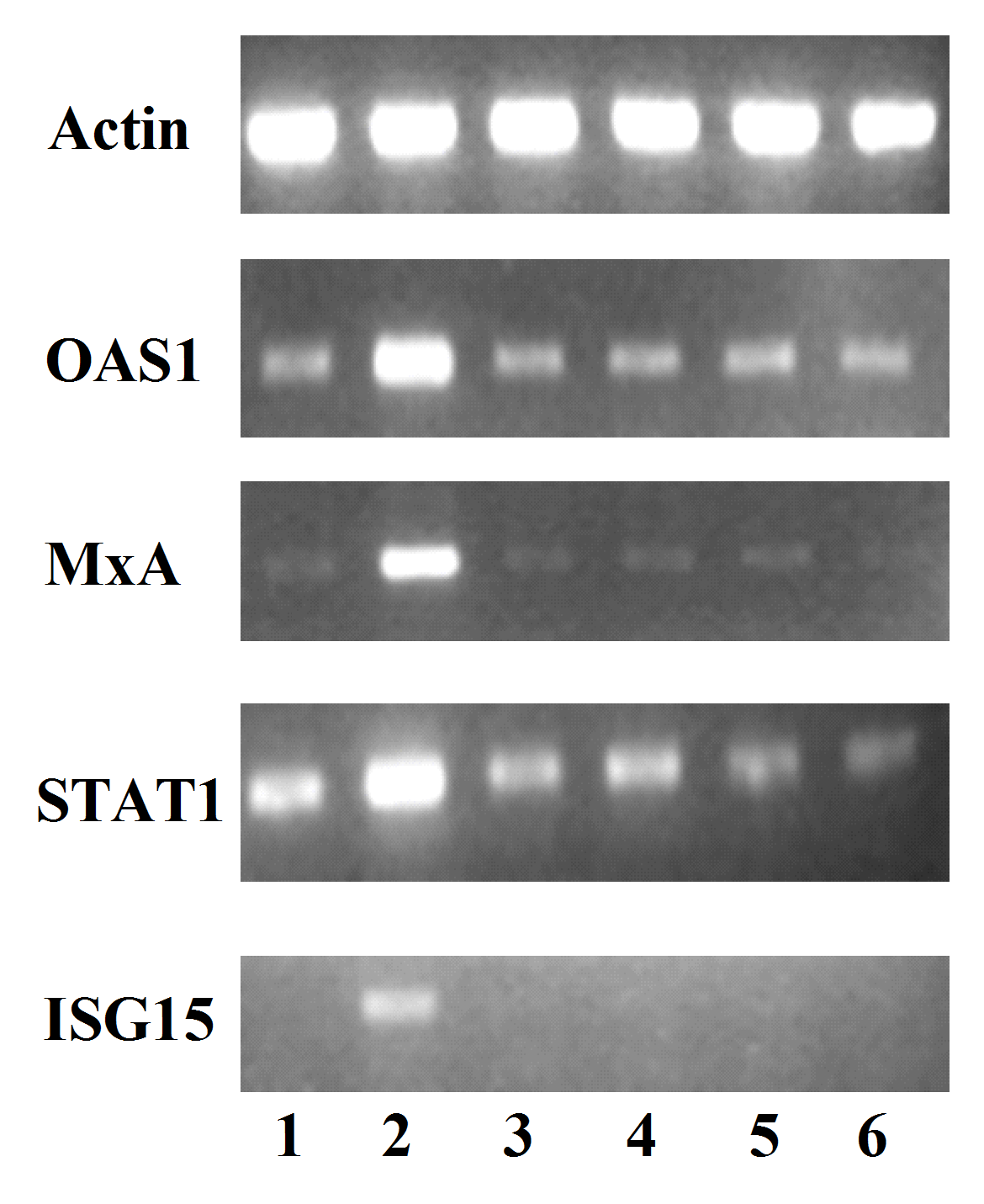

Supplement: Figure S5 — Transfection with shRNA vectors didn't induce an obvious interferon response. Lane 1, mock; lane 2, IFNα-2a; lane 3, AS139; lane 4, AS1819; lane 5, AS3172; lane 6, neg. (TIF) [file pone.0056110.s005.tif]

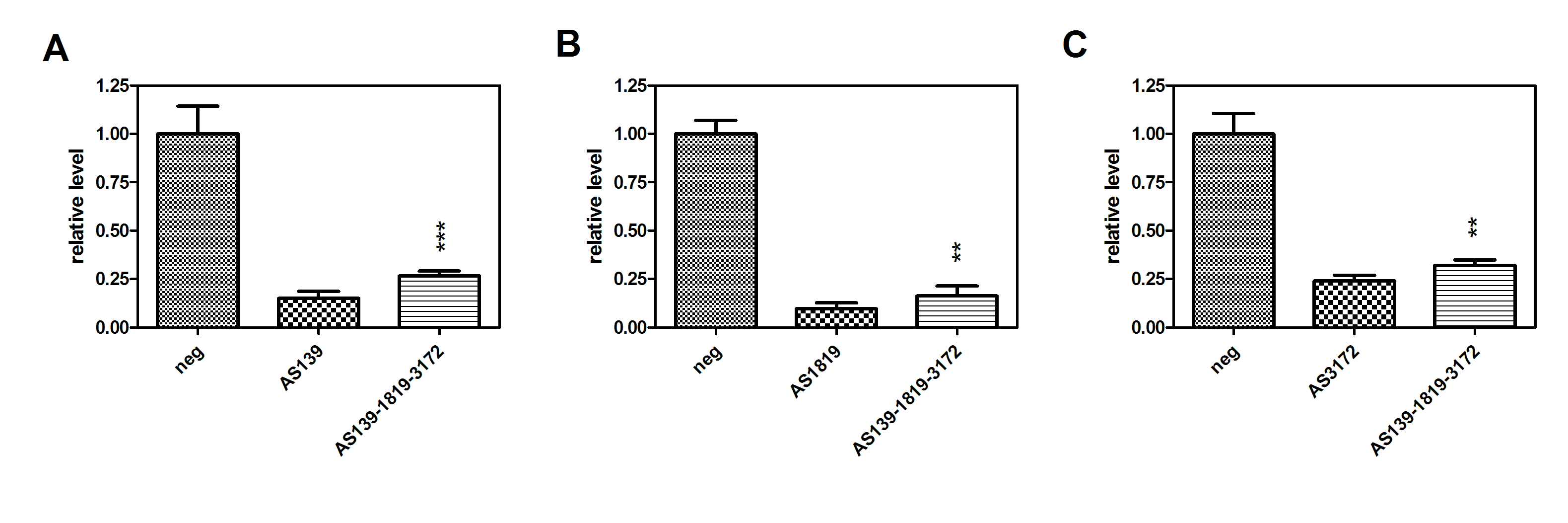

Supplement: Figure S6 — An indirect experiment using the 3'-UTR luciferase assay to demonstrate that these shRNAs were expressed. The relative luciferase level of AS139-1819-3172 was compared with AS139, AS1819 and AS3172 respectively. (TIF) [file pone.0056110.s006.tif]

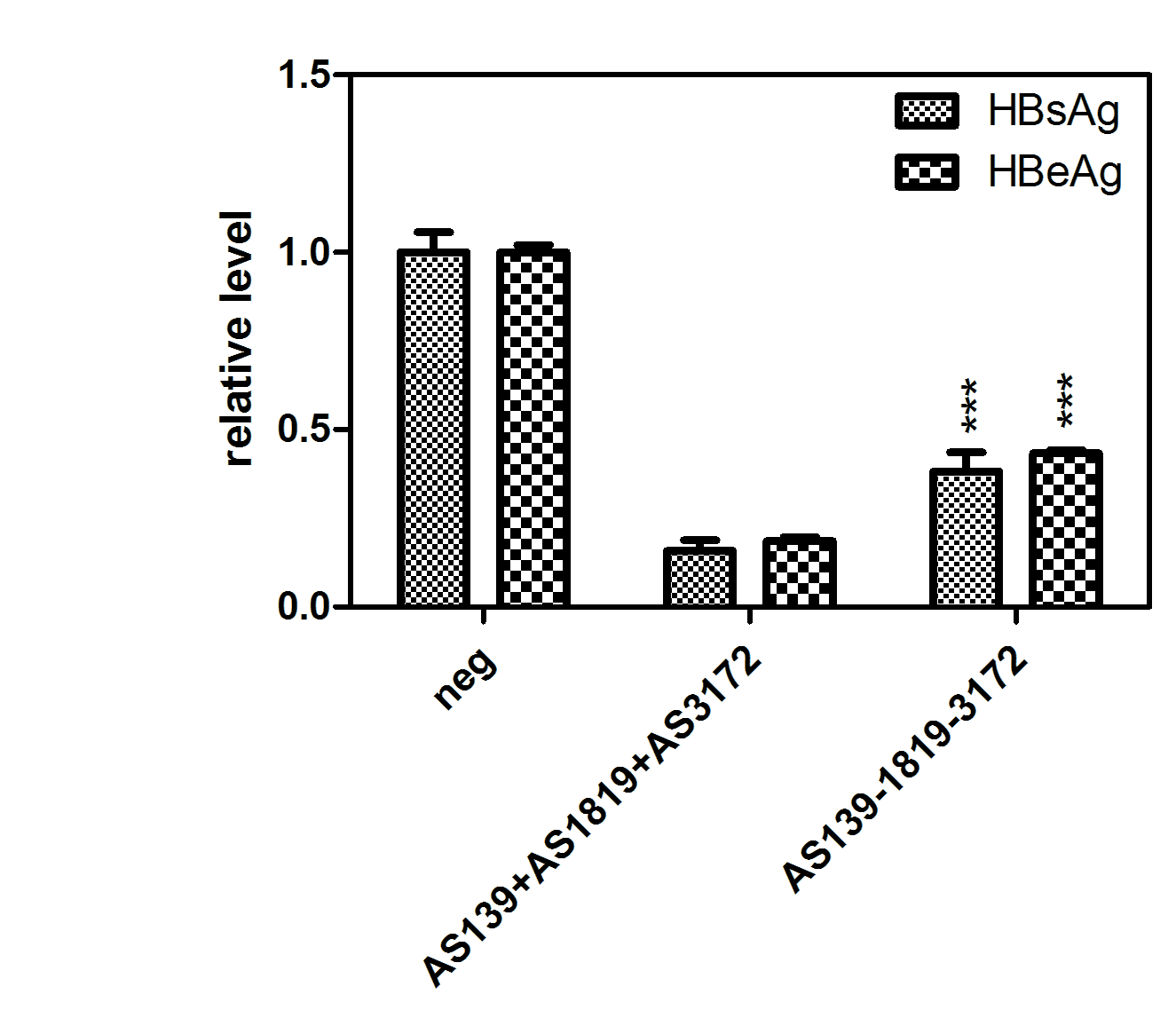

Supplement: Figure S7 — The co-transfected multiple shRNA constructs had better silencing activity than the shRNA plasmid with multiple shRNAs when the same amount of each shRNA scaffold was used. (TIF) [file pone.0056110.s007.tif]
